# Copy Number Variations in Neuropsychiatric Disorders

**DOI:** 10.3390/ijms241813671

**Published:** 2023-09-05

**Authors:** Gergely Büki, Kinga Hadzsiev, Judit Bene

**Affiliations:** Department of Medical Genetics, Clinical Center, Medical School, University of Pécs, 7624 Pécs, Hungary; buki.gergely@pte.hu (G.B.); hadzsiev.kinga@pte.hu (K.H.)

**Keywords:** psychiatric disorder, schizophrenia, bipolar disorder, major depressive disorder, CNV, copy number variation

## Abstract

Neuropsychiatric disorders are complex conditions that represent a significant global health burden with complex and multifactorial etiologies. Technological advances in recent years have improved our understanding of the genetic architecture of the major neuropsychiatric disorders and the genetic loci involved. Previous studies mainly investigated genome-wide significant SNPs to elucidate the cross-disorder and disorder-specific genetic basis of neuropsychiatric disorders. Although copy number variations represent a major source of genetic variations, they are known risk factors in developing a variety of human disorders, including certain neuropsychiatric diseases. In this review, we demonstrate the current understanding of CNVs contributing to liability for schizophrenia, bipolar disorder, and major depressive disorder.

## 1. Introduction

In the past 20 years, many large-scale genetic studies have been published on the genetic basis of neuropsychiatric disorders and their development. Neuropsychiatric disorders, such as schizophrenia (SCZ), bipolar disorder (BD), major depressive disorder (MDD), intellectual disability (ID)/developmental delay (DD), autism spectrum disorder (ASD), attention deficit hyperactivity disorder (ADHD), Tourette syndrome (TS), and obsessive compulsive disorder (OCD), are complex conditions which represent a significant global health burden with complex and multifactorial etiologies. Most of them are at least moderately inherited [1]. Based on twin studies, the estimated heritability of SCZ, BD, and ADHD is around 75%–80% [2,3,4]; however, the heritability of MDD is much lower, with ca. 40% [5]. The etiology of neuropsychiatric disorders might be affected by the cumulative impact of multiple factors and certain gene-environment interactions.

**Figure 1:**
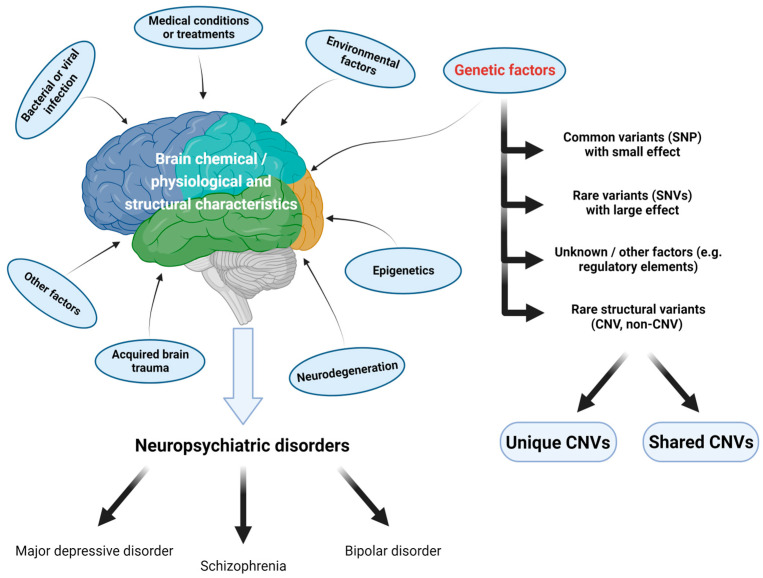
Associations in the development of neuropsychiatric disorders. The figure was created via BioRender.com (accessed on 23 August 2023).

Technological advances in recent years have improved our understanding of the genetic makeup of the major neuropsychiatric disorders and the genetic loci involved [1]. Other than common low-risk single nucleotide polymorphisms (SNPs) [6,7] and noncoding RNAs [8], more and more studies suggest that copy number variations (CNVs) contribute to the susceptibility of neuropsychiatric disorders and they are identified as crucial contributors to the pathogenesis of such diseases [9,10,11] (Figure 1). Although most of them focused on disorder-specific CNV identification, substantial progress has been made in unraveling the intricate relationship between neuropsychiatric disorders and CNVs.

A copy number variation is a type of structural variant appearing quite often in an individual’s genome. CNVs are large DNA segments that are present at variable copy numbers compared to the reference genome (resulting in the deletion, duplication, or amplification of a certain DNA region) [12]. CNVs represent a major source of genetic variations widely distributed across the genome and are known risk factors in developing a variety of human disorders [13,14]. Moreover, the de novo locus-specific mutation rates appear much higher for CNVs than for SNPs [15,16,17]. CNVs present approximately 12–16% of the human genome, comprising both population-specific and individual-specific variation. It has been revealed that three to seven rare CNVs can be found in an average genome [18]. Among them, deletions are less common than duplications [19]. Moreover, CNVs larger than 500 kb in size can be found in 5% to 10% of the population while 1% to 2% of the individuals have CNVs greater than 1 Mb in size [18]. Based on the variant allele frequency (VAF), CNVs can be common (VAF ≥ 1%) or rare (VAF ≤ 1%). The frequency of a CNV shows a strong anticorrelation with its size and its gene density [20]. Moreover, CNVs can be classified as benign or pathogenic according to their impact on human diseases. Benign CNVs are frequently small, intergenic, or comprise genes that can tolerate copy number changes. Pathogenic CNVs are significantly enriched for genes involved in development and genes with constrained evolutionary patterns of gene duplication and loss [21].

Germline CNVs are supposed to be responsible for the missing heritability in complex diseases and traits after receiving unexpected results from of genome-wide association studies [22]. Several attempts have been made to explore the association between CNVs and various complex diseases and they led to the conclusion that common CNVs do not account for the missing heritability after GWAS for complex traits. In contrast, several rare CNVs have been implicated in the development of various diseases [23]. In comparison with neuropsychiatric disorders, a relatively small number of studies have explored the association between CNVs and other common complex diseases, such as diabetes, cancer, and cardiovascular disorders [24,25,26,27,28]. Even among these, only a few examine the effect of CNVs on one gene [24,25] or analyze subpopulations [26], for instance, in studies on type-2 diabetes. Although, a growing number of studies provide further insights into the role of CNVs in the pathogenesis of common complex disease traits, as presented in the work of Glessner et al. [28].

CNVs may lead to dosage imbalances and alter expression levels, which influence human phenotypes. CNVs may affect specific genes that are essential for neurodevelopmental processes, synaptic function, and neuronal signaling pathways; changes in their dosage may contribute to the disruption of crucial molecular pathways associated with neuronal function and mood regulation [17]. It has been observed that individuals with neuropsychiatric disorders exhibit a higher burden of rare and large CNVs compared to healthy controls [13,29].

**Figure 2:**
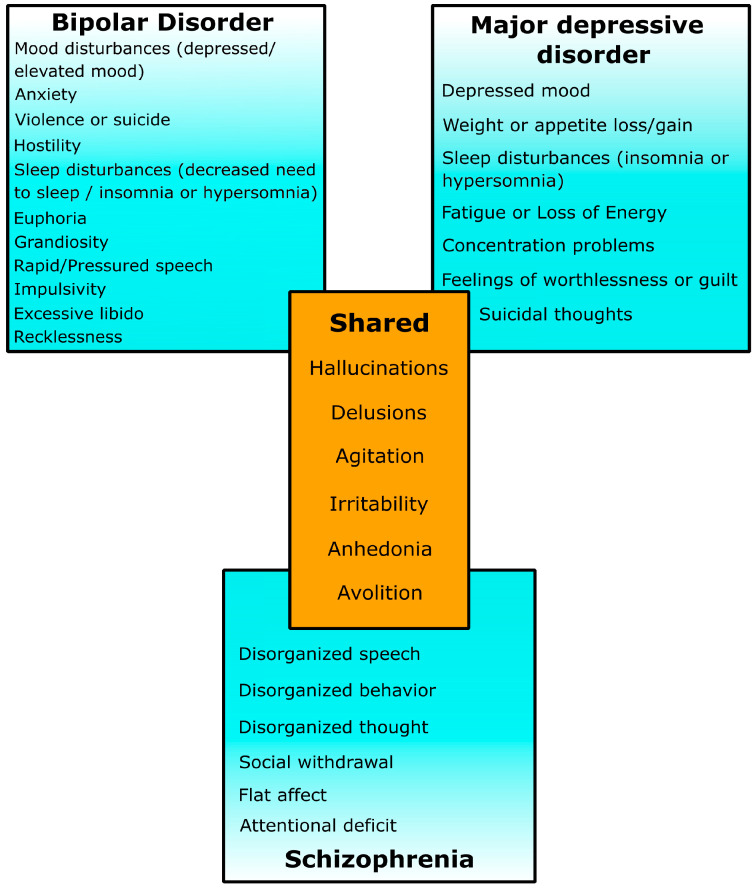
Clinical manifestations of schizophrenia, bipolar disorder, and major depressive disorder, according to the Diagnostic and Statistical Manual of Disease (5th edition) [30].

In this review, we will discuss the current knowledge of CNVs contributing to liability for neuropsychiatric disorders. We focus on three major psychiatric disorders, namely, SCZ, BD, and MDD, which are distinct disorders with their own specific features and treatment approaches; however, they share some common behavioral characteristics and cognitive deficits (Figure 2). Moreover, they share genetic liability and underlying molecular mechanisms.

## 2. CNVs in Schizophrenia

Schizophrenia is a complex, clinically heterogeneous mental disorder with a prevalence of approximately 1% and a shorter life expectancy than the general population [31,32]. It is primarily characterized by disordered thinking, delusions, emotional deficits, and hallucinations. The onset generally occurs during late adolescence or early adulthood; although, rare cases have been presented with onset during childhood [32]. The aetiology of schizophrenia is thought to be multifactorial, based on complex interactions between genetic and environmental factors that influence early brain development and the trajectory of biological adaptation to life experiences [19,33]. Among the genetic variations described in patients with schizophrenia so far, CNVs are one of the most frequent genetic variations that might be causative [19].

The first reviews on the relationship between psychiatric disorders and chromosomal abnormalities were published in the early 1980s, including a study on chromosomal abnormalities associated with schizophrenia-like psychoses by Propping (1983) [34] and a study on fifteen X-linked and four autosomal abnormalities associated with psychoses by DeLisi and Lovett (1990) [35]. Previous research from family, twin, and adoption studies has shown strong evidence that genetic vulnerability underlies SCZ [36,37,38]. The first CNV associated with increased risk for schizophrenia/schizoaffective or bipolar disorder was the 22q11.2 deletion [39,40,41,42,43]. Individuals with the 22q11.2 deletions have a 20-fold increased risk of schizophrenia [44,45]. The deleted region contains genes that have been implicated in the aetiology of idiopathic psychosis [46,47]. According to Bassett et al., individuals carrying the 22q11 deletion have a highly variable phenotype and the average age at death is around their 40s [45]. Nearly 25% of carriers of the 22q11.2 microdeletion have schizophrenia-like symptoms [48]. In addition, reduced risk of SCZ has been associated with duplications of 22q11.2 [49].

The role of CNVs in the development of and/or contribution to schizophrenia is beginning to unfold [14,50,51] and it has been proposed that the CNV burden is higher in schizophrenia cases than in controls [50,52,53,54]. Remarkable differences in CNV burden have been observed between females and males in the general population and, also, in patients suffering from neurodevelopmental disorders, such as schizophrenia [55]. However, Han and colleagues demonstrated that variation in the gender CNV burden has no effect on known schizophrenia–CNV associations [29].

The genome-wide enrichment of rare duplications and deletions and the higher rates of de novo CNVs in SCZ cases compared to controls, as well as associated evidence, are shown to play a role in a number of specific loci, all contributing to the risk of SCZ development. According to a previous analysis of 15 schizophrenia-associated loci by Rees et al., higher rates were found in the case group compared to the control group in 13 of the 15 CNV-examined loci. Strong evidence has been demonstrated for the association between an increased risk of schizophrenia and 11 CNVs. The remaining four CNV loci represented weaker evidence and required further investigation [56]. The association with schizophrenia is now well established, accounting for approximately 1.0% of schizophrenia cases in the general population [11]. Former studies reported a higher de novo CNV mutation rate in SCZ in cases lacking a family history [57,58]. The results presented by Rees et al. demonstrated that circa 2.5% of individuals with SCZ carry at least one known pathogenic CNV [29,56].

Although most rare CNVs implicated in schizophrenia are unique and occur de novo, several were observed at recurrent genomic hotspots. This originates from the genomic architecture of hotspots, which are typically characterized by large repeated segments that increase the risk of replication errors and ultimately lead to the formation of CNVs [59]. Such genomic hotspots occur at the chromosome regions of 1q21.1, 3q29, 15q11.2, 15q13.3, 16p11.2, 16p12.1, 16p13.11, 17p12, and 22q11.2 and the neuropeptide receptor VIPR2. These loci have been associated with SCZ [59,60]. Although, further meta-analysis proved that the association between SCZ and *VIPR2* is no longer supportive [56]. In a large, genome-wide study, Marshall et al. obtained definitive evidence of association for at least eight CNV loci in schizophrenia. Moreover, they found suggestive support for eight additional candidates for susceptibility and protective loci [11]. Based on previous findings, microduplications of 22q11.2 appear to confer protection from SCZ [49]. In other studies, strong statistical evidence has been proven for 12 risk CNVs associated with schizophrenia [13,51,61]. CNVs strongly associated with SCZ, based on large case-control studies, are presented in Table 1 and Table 2. Moreover, Table 3 and Table 4 demonstrate statistical data related to the unique and shared CNVs.

In a cohort of patients with schizophrenia, Hubbard et al. [68] investigated the cognitive ability between known schizophrenia-associated CNV carriers and noncarriers; they found that individuals harboring CNVs associated with SCZ exhibit significant cognitive impairment with clinical relevance. Furthermore, rare CNVs affecting gene sets (loss-of-function intolerant and synaptic gene sets) enriched for SCZ are specifically linked to poorer cognitive function in individuals with SCZ. Their results suggest that cognitive impairment could be used to prioritize patients for CNV testing [68]. Yeo et al. (2021) demonstrated that a greater number of uncommon deletions are associated with larger ventricle size and reduced general cognitive abilities in patients with schizophrenia compared to healthy controls. They suggested that gray matter volume did not interfere with the relationship between overall cognitive ability and deletion burden [69].

Previously, the majority of CNV analysis in schizophrenia has focused on disorder-specific CNV identification and only a few studies have investigated the effect of CNVs on specific sub-phenotypes of schizophrenia. In their study, Merikangas et al. aimed to explore the association between genome-wide CNVs and risk factors and subphenotypic features of SCZ beyond cognitive function. They examined the clinical outcome of CNVs that impacted differentially brain-expressed genes in a case-only analysis with 386 cases. Their results did not reveal any statistically significant association between CNVs impacting differentially brain-expressed genes and clinical severity, developmental delay, pre-morbid intelligence and intelligence quotient, and social cognition phenotypes. However, they found that advanced paternal age is associated with deletions in brain-expressed genes and brain-expressed CNVs are less common among those with a positive family history of psychiatric disorders. Based on their findings, the authors hypothesized that CNVs may represent one pathway to schizophrenia while a positive family history may indicate other genetic mechanisms that increase vulnerability to schizophrenia [52].

Larger (>500 kb) exonic CNVs have been shown in some research studies to have the highest burden in schizophrenia and they might have an effect on the phenotype in patients at risk of schizophrenia. Schizophrenia might be the only phenotypic feature of patients carrying CNVs; in other cases, schizophrenia is associated with intellectual disability or it develops as part of known genetic syndromes attributable to CNVs [19], such as 1q21.1 microduplication/microdeletion syndrome [70], Burnside–Butler syndrome (15q11.2 microdeletion) [71], or 22q11.2 microdeletion syndrome [44]. Based on previous studies, it appears that patients with schizophrenia who have an associated intellectual disability are more likely to carry pathogenic CNV [46,72,73]. Thygesen et al. found around a threefold higher frequency of pathogenic CNVs in individuals with psychosis and an intellectual disability than in the general schizophrenia population [72]. Moreover, Lowther and colleagues demonstrated how there is a significantly higher burden of pathogenic CNVs among patients with schizophrenia and a low IQ (IQ < 85) compared with those with an average IQ (IQ ≥ 85) [73]. These findings implicate that individuals with schizophrenia and a low IQ should be prioritized for clinical microarray testing in clinical and research contexts [31,72,73]. Derks et al. [74] found that in the case of very large CNVs (>1 Mb), the rates of CNVs were increased in cases with an ID and SCZ compared to ID-only cases. However, no significant differences were found in cases with CNVs smaller than 1 Mb. Based on their findings, the authors hypothesized that an ID plus SCZ is a severe form of SCZ, instead of a separate diagnostic category or a comorbid condition [74]. Rees and colleagues investigated the burden of 51 CNVs implicated in IDs for association with SCZ and 12 known schizophrenia-associated CNVs in a large case-control study. They found that a large proportion of the ID loci are likely to be risk factors for SCZ. Their findings indicate that a larger sample size will identify additional ID CNVs that are true SCZ risk factors. In addition, their results support the evidence for an aetiological overlap between neurodevelopmental disorders. A known ID-associated CNV occurring in a patient with SCZ should raise the suspicion that it is relevant to the psychiatric disorder in that individual [75].

Although SCZ commonly emerges during the late adolescence and early adulthood stages, in rare, severe cases, it might occur with onset during childhood. It is usually defined before the age of 13 years. Approximately 4% of patients encounter early-onset schizophrenia (EOS), which manifests either during childhood, before the age of 13, or during adolescence up to the age of 17 years [76]. It is suggested that a higher genetic load and increased symptom severity are observed in EOS. Familial investigations involving patients with childhood-onset SCZ (COS) reveal an increased rate of schizophrenic spectrum disorders pointing to familial transmission [77]. A previous study on patients with COS, adult-onset SCZ (AOS), and ancestry-matched controls presented that a higher percentage of patients with AOS (15%) exhibited novel structural variants compared to the controls (5%) [78]. In contrast, 28% of patients diagnosed with COS and 20% of patients with onset of SCZ before 18 years old carried one or more rare SVs [77]. In addition, another study provided evidence that 10% of individuals with COS demonstrated large chromosomal abnormalities at higher significant rates compared to patients with AOS [79]. COS patients also carried additional genomic lesions at 8q11.2, 10q22.3, 16p11.2, and 17q21.3, which have been previously connected with other disorders (e.g., autism, intellectual disability) [77]. Ahn et al. conducted whole genome genotyping, mainly focusing on 46 rare cases of CNVs, which have well-known risks for adult-onset SCZ. Compared to AOS, COS displayed a higher rate. Moreover, a higher rate was observed in cases of 22q11.2 deletion. Altogether, their observations provide support for the widely recognized pleiotropic impacts of these CNVs indicating shared abnormalities early in brain development. Among 126 COS patients, they discovered that 15 COS probands (11.9%) had at least one disease-related CNV. CNVs that were associated with SCZ included the deletions of 2p16.3, 15q11.2, 15q13.3, and 22q11.2. and duplications of 2p25.3 and 16p11.2 [32]. Consequently, patients diagnosed with COS are anticipated to exhibit more noticeable neurodevelopmental deviations compared to individuals with AOS [77].

Strong associations established between CNVs and psychiatric disorders are based on multicenter studies covering various populations. However, revealing the population-based prevalence and risk is crucial for determining the individuals’ CNV statuses in order to tailor precision health care. Sanchez et al. provided findings regarding the population-based prevalence and risk of psychiatric disorders associated with pathogenic CNVs in the Danish population. They examined six genomic loci (1q21.1, 15q11.2, 15q13.3, 16p11.2, 17p12, and 17q12) and, compared with previous studies, they found lower risk estimates of SCZ for most CNVs [62]. In another study of the Danish population, Olsen and colleagues found an elevated risk of 22q11.2 loci for SCZ; but, it was considerably lower than expected from previous studies [80]. Kushima et al. [81] performed a comparative CNV analysis on SCZ and ASD cases and found an increased genome-wide burden of rare exonic CNVs. They also identified 12 novel genes in the Japanese population that are potentially associated with SCZ/ASD. Kushima and colleagues in another study conducted an extensive, genome-wide CNV analysis within a predominantly Japanese population. Their investigation verified a significant correlation between X-chromosome aneuploidies with SCZ and 11 de novo CNVs [82]. Zhao et al. performed an investigation of the role of rare CNVs at 15q11.2 in SCZ in the Han Chinese population. A three-fold increase of deletions in cases over controls was found, which suggests an association between CNVs at 15q11.2 and SCZ in the Chinese Han population [83]. Furthermore, Saxena et al. analyzed the 15q11.2 loci on the Indian population and found no significant difference in the frequencies among the controls and patients; however, the sample size was limited [84]. Li et al. [85] examined CNV loci in a Han Chinese population. An increased genome-wide CNV burden was confirmed, which was even more significant when only >1 Mb CNVs were considered. In addition, duplications at 1p36.32, 10p12.1, and 13q13.3 were displayed as new potential loci. Priebe et al. [86] did not find a significant association between specific CNVs and SCZ in the German population when performing a genome-wide analysis. Moreover, the analysis of 11 schizophrenia-associated CNVs revealed no significant association with SCZ. In addition, CNVs in 3q29, 15q13.3, 17p12, or 17q12 did not occur in this patient cohort.

## 3. CNVs in Bipolar Disorder

Bipolar disorder, previously known as manic depression, is a complex psychiatric condition with a high degree of phenotypic heterogeneity. It is characterized by recurrent episodes of depression alternating with periods of hypomania and/or mania, which affect thought, perception, emotion, and social behavior. The severity or magnitude of manic episodes can exhibit considerable variation and milder (hypo)manic episodes can occur in 2–3% of the general population [87]. BD can generally be divided into two subtypes: BD-I (characterized by episodes of mania and depression) and BD-II (hypomania and depression). BP is one of the leading causes of disability worldwide [88]. The observed lifetime prevalence of BD is approximately 2.4%, with an incidence of 0.6% for bipolar type I and 0.4% for bipolar type II [88]. The estimated heritability is around 60–85% [89]. The aetiology of BD is not well understood; however, due to intensive research over the last decade, more and more evidence has been revealed regarding the genetic background of the disorder, risk and vulnerability factors, and gene–environment interactions.

Although CNVs have been less well studied among the genetic factors in BD, certain CNVs have been reported to be associated with BD [90]. Zhang et al. investigated the frequency of singleton CNVs, which occur once in a dataset, among individuals with BD. These rarest CNVs are of interest since they are presumably enriched in de novo events. Singleton deletions over 100 kb in length throughout the genome were reported to be more frequent in BD cases compared to healthy controls; therefore, they are supposed to increase the risk of bipolar disorder [91]. The enrichment of de novo CNVs in individuals with BD was presented by Malhotra and colleagues as well [92]. In their trio-based study, 10 CNVs were identified as having a 5.4% rate in BD patients compared to a rate of 0.9% in the controls. In addition, Noor et al. found eight de novo CNVs, which were represented in a rate of 3.7% among BD individuals [93]. In a subsequent study, Georgieva et al. investigated the role of de novo CNVs in the aetiology of BD and compared them with de novo CNVs detected in SCZ. The presence of 15 de novo CNVs was revealed in BD, with a frequency of 4.1%. After combining their results with previous studies, they found that the increase in the de novo rate in BD over the controls was not significant. Compared to SCZ, the association between de novo CNV variants, especially large and rare CNVs, and BD seems to be more modest. In addition, de novo CNVs tend to be smaller in BD patients compared to SCZ [94].

Green et al. examined fifteen CNV loci previously known to be associated with SCZ and observed that three of them (16p11.2 and 1q21.1 duplications, 3q29 deletion) displayed significantly higher rates among BD patients compared to the control group. The 16p11.2 duplication showed the strongest evidence for association [64]. Their results confirmed that a significant difference is observed between BD and SCZ in terms of CNV occurrence, in particular, for large deletions exceeding 1 Mb in size. A pilot study by Chen and colleagues on the commonality and specificity of small common, and large CNVs in SCZ and BD demonstrated that the 10q11.21-22 variant, which affects the *GPRIN2* gene involved in neurite outgrowth, is associated with an elevated risk of BD. In addition, variants in the 17q21.2, 9p21.3, and 9q21.13 regions displayed BD associations only. However, the 11p15.4, 15q13.2, and 22q11.21 variants are likely SCZ-specific, without BD associations [95].

The age at onset is often taken into account in CNV investigations. Some studies divided BD individuals into early- or late-onset subgroups, depending on the age of onset (AO) of the diagnosis [92,96]. Compared with healthy controls, in the patient cohort with an AO ≤ 18, the rate of de novo CNVs was significantly higher [92]; meanwhile, a higher frequency of microduplication CNVs and a significantly larger average size of singleton microdeletions were observed in patients with an AO ≤ 21 [96]. Two common CNV loci, microduplications on 6q27 and on 10q11, were enriched among early onset BD patients. The findings of Priebe et al. suggest that CNVs have an impact on the development of early-onset but not later-onset BD [96].

Although a number of singleton CNVs have been identified in association with BD, only a few recurrent CNVs have been confirmed in large case-control studies. These CNVs are displayed in Table 1 and Table 2.

The identified CNVs in BD patients implicate a number of candidate genes involved in biological processes crucial to normal human development. The disruption of these gene functions is supposed to be involved in the pathogenesis of BD. The *GRIK2* gene is located in the 6q16.3 CNV region, which is essential for brain development [97,98]. Noor et al. reported several CNVs that affect synaptic protein-encoding genes, such as *DLG1*, *DLG2*, *DPP6*, *NRXN1*, *NRXN2*, *NRXN3*, *SHANK2*, and *EPHA5*; neuronal splicing regulator genes, such as *RBFOX1*; and the *CHL1* gene, which encodes a neuronal cell adhesion molecule.

## 4. CNVs in Major Depressive Disorder

Major depressive disorder is a mental health condition with a lifetime incidence of 10–20% [99]. It is characterized by a persistently low or depressed mood and a loss of interest in activities that used to bring pleasure. It can have a significant impact on individuals’ personal, social, and occupational functioning and affect quality of life. The morbidity from depressive perceptions and mortality from suicide attempts are substantial. Compared to several other psychiatric disorders, the heritability in MDD is lower, at around 37% [100]. The development of MDD is thought to be affected by a complex interaction of genetic, environmental, and neurobiological factors [101].

Based on initial research, no significant genome-wide CNV burden was observed in MDD [102]. Glessner et al. reported the first genome-wide association analysis of CNVs in MDD. The most significant locus was in the chromosomal region 5q35.1. Microduplications on 5q35.1 harbor the *SLIT3*, *CCDC99*, and *DOCK2* genes, of which, *SLIT3* is involved in axon development [100]. In their study, Degenhardt and colleagues found microdeletions in 7p21.3 and 18p11.32, microduplications in 15q26.3, and the combination of microdeletions/duplications in 16p11.2 to be more frequent in MDD patients compared with controls [103]. O’Dushlaine and colleagues investigated the association of rare CNVs with treatment-resistant MDD. They found that the contribution of rare CNVs to treatment-resistant depression appears to be modest [102]. A meta-analysis was carried out by Zhang et al. and demonstrated a notable increase in rare, short (<100 kb) deletions in MDD cases [101]. However, long, multigenic CNVs are potentially less likely to have significant effects on the risk of MDD. Based on these results only short deletions (10–100 kb) show a significant effect on MDD risk; however, the analysis of larger cohorts might provide more significant results about longer deletions [101]. As the observed deletions are particularly intergenic, it was hypothesized that their impact on MDD risk is due to the deletion of regulatory elements [101]. A higher prevalence of mood disorders has been observed in individuals with 15q13.3 duplications compared to individuals with reciprocal deletions (21.5% and 12%, respectively) [104]. According to Gillentine et al. [105], dosage changes in the *CHRNA7* gene may be relevant to MDD and anxiety disorders. They observed a four-fold increase in the rate of *CHRNA7* gain among individuals (adolescents, emerging adults) with MDD and anxiety disorders. A copy number polymorphism within the 10q11.21 region might be involved in the genetic etiology of suicide attempts in MDD, as Shitao Rao and colleagues suggested [106]. It potentially affects the expression of *ZNF33B* in the brain [106].

The role of CNVs in response to treatment with antidepressants was investigated in a study by Tansey et al. among individuals with MDD [107]. No significant association was observed between the antidepressant response and the presence of CNV, the overall number of CNVs, or the genomic CNV burden. However, a nominally significant association was seen with specific CNVs, such as exonic deletions of *NRXN1* and duplications of 15q13.3. Notably, individuals harboring these CNVs exhibited a poorer response to antidepressant medications. A comparative study on the Danish population represented that the 1q21.1 deletion is associated with increased risk for MD and, exclusively in males, for BD [62].

**Figure 3:**
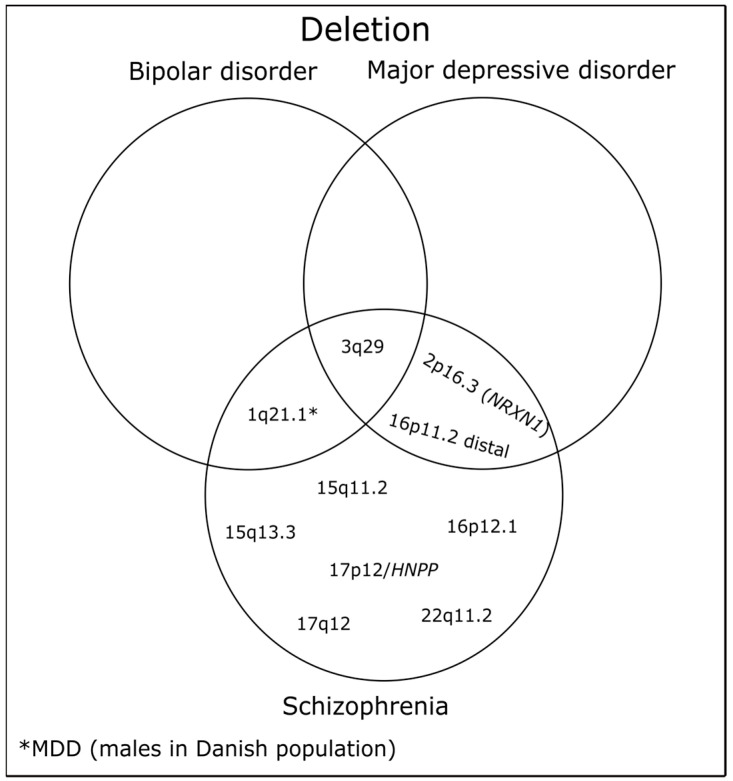
Representation of unique and shared deletions among MDD, SCZ, and BD.

**Figure 4:**
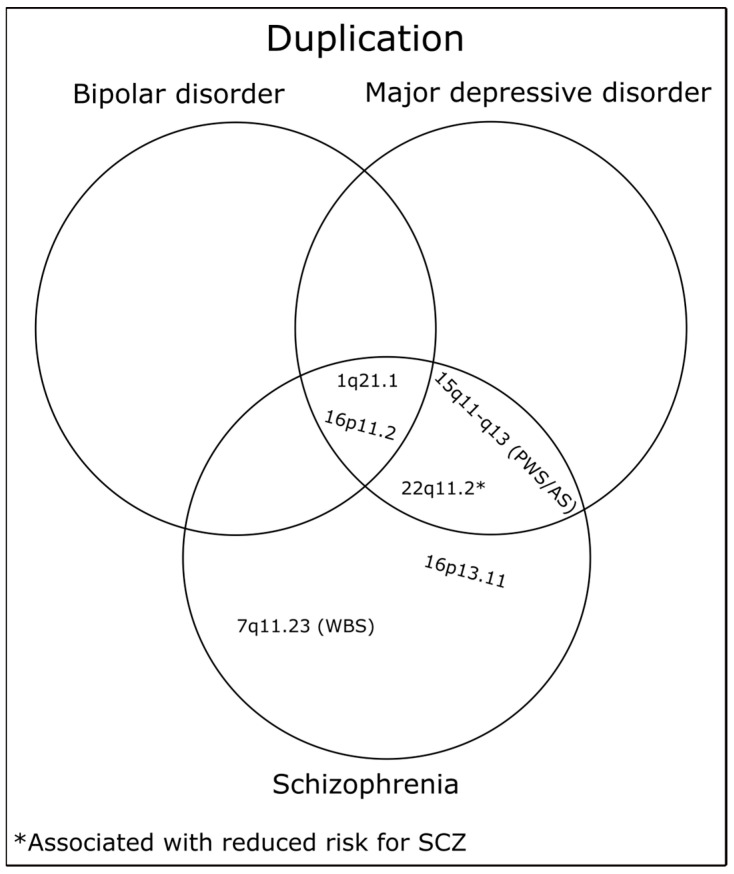
Representation of unique and shared duplications among MDD, SCZ, and BD.

CNVs strongly associated with MDD based on large case-control studies are presented in Table 1 and Table 2. Some CNVs overlap between MDD, SCZ, and BD, which are displayed in Figure 3 and Figure 4.

## 5. Tools for Detection and Investigation of the Biological Significance of CNVs

The screening and characterization of CNVs are challenging tasks because of their genomic complexity and technical limitations. After the initial analysis of the entire chromosomes, numerous molecular diagnostic methods have been developed and applied in the past years. As techniques have evolved, the resolution of detection improved over time. Currently, array-comparative genomic hybridization (CGH), SNP-based microarray techniques, and, lately, short-read sequencing are the most frequently applied approaches. The usage of long oligonucleotide arrays facilitated a more accurate characterization of CNVs and provided a more detailed analysis of the genome. Improvements provided a better signal-to-noise ratio and a more complete genome coverage and assisted the process of chip development.

Several algorithms and software packages have been created to identify CNVs and all of them have their advantages and limitations [23,108,109,110]. Zhang and colleagues compared the performance of four software packages and reported that PennCNV is superior in terms of specificity and sensitivity compared to the others [111,112]. As time goes by, newer algorithms and software are being developed, for example, EnsembleCNV, which is a novel ensemble learning framework used to detect and genotype CNVs [113].

Although early usage of high-throughput sequencing was based on the detection of SNPs and small indels, the advancements in methodology and bioinformatics helped the application of next-generation sequencing (NGS) in CNV detection. Short-read sequencing technologies with a large number of overlapping fragments provide more accurate breakpoint resolution of CNVs and are capable of identifying smaller CNVs compared to the microarray technologies. Despite many different algorithms, pipelines, and methodologies being developed, such as paired-end mapping, split-read methods, read-depth methods and de novo assembly, the proper detection of CNVs is still a challenging task. As of now, the most accurate approach to determine CNVs is complete genome assembly compared to a high-quality reference genome. Whole-exome sequencing (WES) is frequently preferred due to its cost-effectiveness compared to whole-genome sequencing (WGS). Although, the targeted regions in WES are quite short and discontinuous across the genome. In the future, the application of longer-read technologies and newer algorithms can potentially improve the characterization of CNVs.

Lately, a new technology known as optical genome mapping (OGM) has emerged to diagnose a large variety of chromosomal abnormalities with high accuracy in a cost- and time-effective way. This advanced methodology facilitates the high-resolution reconstruction of the genome by utilizing linearized strands of high molecular-weight DNA. In comparison to the current second- and third-generation sequencing approaches, OGM uses longer DNA sequences to analyze large eukaryotic genomes. OGM is capable of detecting the various types of structural variants, encompassing CNVs.

Long-read single-molecule strategies might overcome previous challenges, such as repetitive sequences and intermediate-size CNVs. Presumably, the long-read sequencing technologies will provide extensive data, which will help to characterize different pathologies connected to CNVs [111].

Furthermore, other approaches are available to examine the underlying pathology of neuropsychiatric disorders, such as investigating induced pluripotent stem cells (iPSCs) from patients with pathogenic CNVs and CNV-based animal models [111,114]. Previously, Nakatani and colleagues [115] reported on a mouse model of the 15q11.2-q13.1 duplication, where mice displayed abnormal social behavior and behavioral inflexibility. In another case, a mouse model of the 16p13.11 duplication, which is suggested as a risk factor for ADHD in addition to schizophrenia, was presented by Fujitani et al. [116], where the model displayed a behavioral hyperactivity phenotype [111].

In addition, iPSCs derived from patients with pathogenic CNVs offer an opportunity for understand the underlying molecular and cellular phenotypes. A promising approach for studying and understanding CNVs associated with neuropsychiatric disorders in the near future is the combination of iPSC-derived neurons and high throughput electrophysiological measurements of neurons [117]. Previous studies examined the expression of miRNAs in neurons from iPSCs. Zhao et al. [118] used iPSCs to examine neuropsychiatric disorders associated with 22q11.2 microdeletions. Toyoshima and colleagues [119] established human iPSCs from two schizophrenia patients with the 22q11.2 deletion. Moreover, the application and examination of human 2D and 3D organoids could provide additional knowledge.

Altogether, human research cohorts, extensive collaborations, neuroimaging studies, and longitudinal evaluations will all be essential to understanding CNV-neuropsychiatric associations in the future [117].

## 6. Biological Impacts of CNVs

Previous studies mainly investigated genome-wide significant SNPs to elucidate the cross-disorder and disorder-specific genetic basis of neuropsychiatric disorders. Population-based analyses decrease penetrance estimates for most studied outcomes and increase incidence estimates for most studied CNVs.

Certain evidence suggests that SCZ, BP, and MDD are monoamine-related disorders that occur in either hypomonoaminergic and/or in hypermonoaminergic states [120]. Specific brain monoamines, such as noradrenaline, serotonin, and dopamine, have been studied in relation to neurological and psychiatric diseases [120]. According to Wyatt et al. [121], reduced monoamine oxidase activity is supposed to be a marker for vulnerability to SCZ. Based on previous findings hypodopaminergic activity is related to the negative symptoms of SCZ while excessive dopamine activity is associated with the positive symptoms [120]. Nielsen et al. [122] demonstrated altered dopamine signaling and psychostimulant sensitivity in a mouse model of SCZ-associated human 1q21.1 microdeletion syndrome. Deficiency of serotonin and noradrenaline are thought to be involved in the pathophysiology of depression [123]. Axons of serotonin and noradrenaline play an essential role in the regulation of the mood [120]. Depressive symptoms are associated with degeneration of monoamine axons in animal models of depression and in patients with depression [120,124,125]. Overall, the pathophysiology of negative/cognitive symptoms in SCZ, BP, and MDD is linked to the degeneration of monoamine axons; meanwhile, the hyper-regeneration of monoamine axons plays a role in the positive symptoms of SCZ.

According to previous research [126], alterations in serotonin system function and the medial prefrontal cortex (PFC), as a result of 2p16.3 (*NRXN1*), may contribute to some of the behavioral alterations; therefore, CNV increases the risk of developing neurodevelopmental disorders. They found, in mice, that reduced Nrxn1α expression induces prefrontal cortex hypometabolism and impairs cognitive flexibility.

The PFC is extensively involved in essential cognitive processes, such as decision making, working memory, and interference control [127]. Individuals with PFC damage exhibit notable deficits in cognitive functioning and memory capabilities [120,128]. A previous study displayed that CNV regions affecting gene expression in the PFC are considerably enriched for rare and gene-harbouring CNVs [129].

Based on all of this, it can be assumed that the gene content involved in the different loci may regulate specific monoamines, affect the development of the prefrontal cortex, or other not yet known factors that may contribute to neuropsychiatric illnesses, especially SCZ, BP, or MDD.

## 7. Conclusions

The presented findings facilitate our understanding of the biological mechanism’s underlying neuropsychiatric disorders and hold potential for improved diagnostic tools, personalized treatment strategies, and the development of targeted therapeutics in the future. Integrating CNV analysis into clinical practice holds promise for improving diagnostic accuracy and facilitating early intervention. Continued research efforts in this field will further elucidate the complex interplay between CNVs and neuropsychiatric disorders, paving the way for precision medicine approaches in the field of psychiatry.

## Figures and Tables

**Table 1:** Different types of deletions associated with neuropsychiatric disorders, as represented in previous case-control and/or family studies, supplemented with the involved genes affected by the deletion size.

Localization	Neurodevelopmental Associations	Position in Mb *	Protein Coding Genes	No. of Genes	Refs.
1q21.1	SCZ, BD, MDD (males in Danish population)	chr1:146.53–147.39	*PRKAB2, FMO5, CHD1L, BCL9, ACP6, GJA5, GJA8*	7	[56,62]
2p16.3 (*NRXN1*)	SCZ, MDD	chr2:50.15–51.26	*NRXN1*	1	[56,63]
3q29	SCZ, BD, MDD	chr3:195.72–197.35	*TFRC, ZDHH19, SLC51A, PCYT1A, TCTEX1D2, TM4SF19, UBXN7, RNF168, SMCO1, WDR53, FBXO45, NRROS, CEP19, PIGX, PAK2, SENP5, NCBP2, NCBP2AS2, PIGZ, MELTF, DLG1, BDH1*	22	[56,63,64]
15q11.2	SCZ	chr15:22.81–23.09	*TUBGCP5, CYFIP1, NIPA1, NIPA2*	4	[56]
15q13.3	SCZ	chr15:31.08–32.46	*FAN1, MTMR10, TRPM1, KLF13, OTUD7A, CHRNA7*	6	[56]
16p12.1	SCZ	chr16:21.95–22.43	*UQCRC2, PDZD9, MOSMO, VWA3A, EEF2K, POLR3E, CDR2*	7	[56]
16p11.2 distal	SCZ, MDD	chr16:28.82–29.04	*ATXN2L, TUFM, SH2B1, ATP2A1, RABEP2, CD19, NFATC2IP, SPNS1, LAT*	9	[56,65]
17p12/*HNPP*	SCZ	chr17:14.16–15.43	*HS3ST3B1, PMP22, TEKT3, CDRT4, TVP23C*	5	[66]
17q12 deletion	SCZ	chr17:34.81–36.20	*ZNHIT3, MYO19, PIGW, GGNBP2, DHRS11, MRM1, LHX1, AATF, ACACA, C17orf78, TADA2A, DUSP14, SYNRG, DDX52, HNF1B*	15	[67]
22q11.2	SCZ	chr22:19.04–21.47	*DGCR2, ESS2, TSSK2, GSC2, SLC25A1, CLTCL1, HIRA, MRPL40, C22orf39, UFD1, CDC45, CLDN5, SEPTIN5, GP1BB, TBX1, GNB1L, RTL10, TXNRD2, COMT, ARVCF, TANGO2, DGCR8, TRMT2A, RANBP1, ZDHHC8, CCDC188, RTN4R, DGCR6L, GGTLC3, TMEM191B, RIMP3, ZNF74, SCARF2, KLHL22, MED15, PI4KA, SERPIND1, SNAP29, CRKL, AIFM3, LZTR1, THAP7, P2RX6, SLC7A4, LRRC74B*	45	[11]

* Genomic localization has been given according to GRCh37 assembly; SCZ, schizophrenia; MDD, major depressive disorder; BD, bipolar disorder.

**Table 2:** Different types of duplications associated with neuropsychiatric disorders, as represented in previous case-control and/or family studies, supplemented with the involved genes affected by the deletion size.

Localization	Neurodevelopmental Associations	Position in Mb *	Protein Coding Genes	No. of Genes	Refs.
1q21.1	SCZ, BD, MDD	chr1:146.53–147.39	*PRKAB2, FMO5, CHD1L, BCL9, ACP6, GJA5, GJA8*	7	[56,63,64]
7q11.23 (WBS)	SCZ	chr7:72.74–74.14	*TRIM50, FKBP6, FZD9, BAZ1B, BCL7B, TBL2, MLXIPL, VPS37D, DNAJC30, BUD23, STX1A, ABHD11, CLDN3, CLDN4, METTL27, TMEM270, ELN, LIMK1, EIF4H, LAT2, RFC2, CLIP2, GTF2IRD1, GTF2I*	24	[56]
15q11-q13 (PWS/AS)	SCZ, MDD	chr15:22.81–28.39	*TUBGCP5, CYFIP1, NIPA2, NIPA1, GOLGA8S, GOLGA6L2, MKRN3, MAGEL2, NDN, NPAP1, SNRPN, SNURF, UBE3A, ATP10A, GABRB3, GABRA5, GABRG3, OCA2, HERC2*	19	[56,63]
16p13.11	SCZ	chr16:15.51–16.29	*BMERB1, MARF1, NDE1, MYH11, CEP20, ABCC1, ABCC6*	7	[56]
16p11.2	SCZ, BD, MDD	chr16:29.65–30.20	*SPN, QPRT, C16orf54, ZG16, KIF22, MAZ, PRRT2, PAGR1, MVP, CDIPT, SEZ6L2, ASPHD1, KCTD13, TMEM219, TAOK2, HIRIP3, INO80E, DOC2A, C16orf92, TLCD3B, ALDOA, PPP4C, TBX6, YPEL3, GDPD3, MAPK3, CORO1A*	27	[56,63,64]
22q11.2	^#^ SCZ, MDD	chr22:19.04–21.47	*DGCR2, ESS2, TSSK2, GSC2, SLC25A1, CLTCL1, HIRA, MRPL40, C22orf39, UFD1, CDC45, CLDN5, SEPTIN5, GP1BB, TBX1, GNB1L, RTL10, TXNRD2, COMT, ARVCF, TANGO2, DGCR8, TRMT2A, RANBP1, ZDHHC8, CCDC188, RTN4R, DGCR6L, GGTLC3, TMEM191B, RIMBP3, ZNF74, SCARF2, KLHL22, MED15, PI4KA, SERPIND1, SNAP29, CRKL, AIFM3, LZTR1, THAP7, P2RX6, SLC7A4, LRRC74B*	45	[56,63]

# Duplication of 22q11.2 is associated with reduced risk for SCZ; * Genomic localization has been given according to GRCh37 assembly; SCZ, schizophrenia; MDD, major depressive disorder; BD, bipolar disorder.

**Table 3:** Statistical data related to the unique and shared CNVs in deletion cases.

	Location	Disease	CNV Frequency (%)		Odds Ratio (95% CI)	*p*	References
			Cases	Controls			
Unique CNVs	15q11.2	SCZ	0.642	0.368	1.8 (1.35, 2.38)	0.000023	[56]
	15q13.3	SCZ	0.098	0.019	4.6 (1.64, 16.22)	0.0015	[56]
	16p12.1	SCZ	0.162	0.045	3.3 (1.61, 7.05)	0.00034	[56]
	17p12/HNPP	SCZ	N/A	N/A	N/A	N/A	[66]
	17q12 deletion	SCZ	N/A	N/A	N/A	N/A	[67]
	22q11.2	SCZ	0.303	0.0049	67.7 (9.3–492.8)	5.70 × 10^−18^	[11]
Shared CNVs	1q21.1	SCZ	0.172	0.026	6.8 (2.9, 18.51)	2.10 × 10^−7^	[56]
		BD	0.033	0.021	1.61 (0.47, 5.5)	0.44	[64]
	2p16.3	SCZ	0.152	0.034	4.5 (2.03, 10.94)	0.000028	[56]
		MDD	0.075	0.037	2.01 (1.18, 3.19)	5.70 × 10^−3^	[63]
	3q29	SCZ	0.069	0.004	18 (2.66, 763.34)	0.0002	[56]
		BD	0.025	0.001	17.31 (1.57, 190.97)	0.03	[64]
		MDD	0.013	0.001	11.22 (2.27, 46.52)	1.00 × 10^−3^	[63]
	16p11.2 distal	SCZ	0.025	0.019	1.7 (0.37, 7.6)	0.51	[56]
		MDD	0.029	0.013	2.23 (0.92, 4.63)	5.00 × 10^−2^	[63]

N/A, not available.

**Table 4:** Statistical data related to the unique and shared CNVs in duplication cases.

	Location	Disease	CNV Frequency (%)		Odds Ratio (95% CI)	*p*	References
			Cases	Controls			
Unique CNVs	7q11.23 (WBS)	SCZ	0.083	0.000	NA	4.00 × 10^−7^	[56]
	16p13.11	SCZ	0.377	0.222	1.7 (1.2, 2.52)	0.0022	[56]
	22q11.2	MDD	0.011	0.063	1.72 (1.12, 2.53)	9.00 × 10^−3^	[63]
Shared CNVs	1q21.1	SCZ	0.108	0.049	2.3 (1.08, 5.09)	0.022	[56]
		BD	0.099	0.037	2.64 (1.19, 5.88)	0.022	[64]
		MDD	0.088	0.040	2.17 (1.34, 3.36)	9.10 × 10^−4^	[63]
	15q11-q13 (PWS/AS)	SCZ	0.083	0.000	NA	4.00 × 10^−7^	[56]
		MDD	0.025	0.003	8.14 (2.77, 21.69)	4.61 × 10^−5^	[63]
	16p11.2	SCZ	0.304	0.030	11 (5.08, 26.43)	3.70 × 10^−15^	[56]
		BD	0.130	0.030	4.37 (2.12, 9)	0.00023	[64]
		MDD	0.071	0.028	2.65 (1.53, 4.31)	2.04 × 10^−4^	[63]

N/A, not available.

## Data Availability

Not applicable.
